# A splendid banana enigma: Phylogenomic assessment of Vietnamese *Musa splendida* and *Musa viridis* populations shows that they are conspecific

**DOI:** 10.1371/journal.pone.0318252

**Published:** 2025-02-11

**Authors:** Yves Bawin, Arne Mertens, Sander de Backer, Dang Toan Vu, Loan Thi Le, Tuong Dang Vu, Steven B. Janssens

**Affiliations:** 1 Meise Botanic Garden, Meise, Belgium; 2 Hasselt University, Hasselt, Belgium; 3 KU Leuven, Leuven, Belgium; 4 Vietnamese Academy of Agricultural Sciences, Ha Noi, Viet Nam; 5 Vietnam National University of Agriculture, Ha Noi, Viet Nam; 6 Université catholique de Louvain, Louvain-la-Neuve, Belgium; Ben-Gurion University, ISRAEL

## Abstract

Species delimitation is essential to study and conserve biological diversity. It is traditionally based on morphological trait variation observed in one or a few specimens. Nevertheless, such assessments may not sufficiently take intraspecific trait variation into account, misidentifying morphotypes as separate species. The use of high-throughput sequencing data alongside morphological data in taxonomic studies may substantially improve the accuracy of taxonomic assessments. The *Musa* genus, commonly known for comprising the wild relatives of banana varieties, consists of about seventy described species. However, the taxonomic status of multiple *Musa* species is uncertain due to typification errors and the lack of high-quality specimens. The species *M*. *splendida* and *M*. *viridis* from northern Viet Nam only substantially differ from each other in the color of their male flower bracts, which is red to pinkish-red in *M*. *splendida* and pink in *M*. *viridis*. Consequently, their taxonomic status as separate species has been debated. Here, we studied the genetic relationships between 121 *M*. *splendida* and *M*. *viridis* plants using high-throughput sequencing data (DArTseq) in which we identified 51,188 single nucleotide polymorphisms. We found that individuals genetically clustered in a principal component analysis (6 clusters), fastStructure analysis (four groups), and ASTRAL-III consensus phylogenetic tree (nine clades) based on their population origin rather than by their taxon identity. In addition, a strong signal for an isolation-by-distance pattern between populations was observed. Plants identified as *M*. *viridis* were more closely related to *M*. *splendida* plants from the same region than to *M*. *viridis* plants from other regions. Hence, we propose to treat *M*. *viridis* as a synonym of *M*. *splendida*.

## Introduction

Species delimitation plays a crucial role in deciphering ecological, biogeographical, and evolutionary patterns across the diversity of life. It is also pivotal for devising effective conservation strategies for endangered species [1]. Traditionally, species have been described based on variation in morphological characters. In plants, some of these morphological characters have a high discriminative power being clearly polymorphic and informative to distinguish among closely related species [2]. However, plants are often characterized by intraspecific morphological variations caused by environmental differences, ontogenetic changes, or genetic mutations [3]. This variability can sometimes lead to misidentification of individuals within the same species as a different species, especially in cases where morphological differences are subtle [2]. Examples of a debatable splitting of species are omnipresent among angiosperm lineages, e.g. *Viburnum* L. (Caprifoliaceae) [4], *Portulaca* L. (Portulacaceae) [5], *Galium* L. (Rubiaceae) [6], *Dianthus* L. (Caryophyllaceae) [7], and Cyperaceae [8]. Conversely, distinct but closely related species with only very subtle morphological differences (for example in only temporarily available generative characters) are sometimes mistakenly classified under the same taxonomic entity [2]. The integration of molecular data alongside morphological variation significantly enhances the accuracy of species identification, particularly in taxa that are subject to uncertainty in their taxonomic status [9]. Recent advancements in high-throughput sequencing have enabled the cost-effective analysis of numerous genomic loci across large sample sizes, revolutionizing taxonomic studies. Despite their high potential to resolve evolutionary relationships among closely related taxa, these methods are not yet standard in supporting species descriptions [10].

Banana (*Musa* L.) is a globally significant food crop, with annual production exceeding 135 million tons in 2022 [11]. Most banana cultivars are derived from intraspecific hybridization events of subspecies of the wild species *M*. *acuminata* Colla, into which other *Musa* species like *M*. *schizocarpa* N.W.Simmonds, *M*. *balbisiana* Colla, and *M*. *textilis* Née were either deliberately or accidentally crossed [12, 13]. About 70 species are described in the *Musa* genus [14]. However, the actual number of *Musa* species remains enigmatic, as the taxonomy of the genus is believed to be confounded by typification errors and difficulties regarding species identification due to the lack of high-quality herbarium specimens and the occurrence of ephemeral flowers [15–17]. Currently, all *Musa* species are categorized into two sections: *Musa* (n = 11) and *Callimusa* (n = 7/9/10) [14]. The wild relatives of cultivated bananas hold potential for crop improvement, yet many are threatened with extinction due to climate change and habitat degradation and adequate conservation efforts are lacking [18]. Wild *Musa* species are found throughout Southeast Asia, spanning from eastern India and southern China to Papua New Guinea and northern Australia [19]. Northern Viet Nam, as part of the northern Indo-Burmese ecoregion [19], is renowned for its rich diversity in wild *Musa* species, with recent surveys identifying six species in the region [20].

Some wild *Musa* species in northern Viet Nam, such as *M*. *splendida* A.Chev. and *M*. *viridis* R.V.Valmayor, Đ.D.Lê & Häkkinen in the *Callimusa* section, exhibit striking morphological similarities. Both species co-occur in the Yên Bái province and are primarily distinguished by the color of the male flower bracts: pink in *M*. *viridis* vs. red to pinkish-red in *M*. *splendida* [21]. Another difference between these species is the shape of the leaf lamina basis: rounded in *M*. *splendida* vs. cuneate in *M*. *viridis*. In contrast to *M*. *splendida*, *M*. *viridis* was reported as non-rhizomatous [22–24]. *Musa paracoccinea* A.Z.Liu & D.Z.Li, endemic to the Yunnan province in southern China, also features red male flower bracts and shares geographic proximity with *M*. *splendida* and *M*. *viridis* [16]. However, *M*. *paracoccinea* plants are considered to be taller (4–6 m vs. 3–4 m), to have ovately shaped male flower bracts (vs. lanceolate-ovately shaped bracts in *M*. *splendida*), and to produce small, bell-shaped seeds (vs. large, bell- or mushroom-shaped seeds in *M*. *splendida*) [23, 24]. It remains unclear whether the morphological differences between these *Callimusa* species justify their separation into separate species [21, 23]. Previous studies using high-throughput sequencing data of *Musa* species have shown the high resolution of these data to discriminate between wild *Musa* species and banana cultivars [12, 25]. Thus, the application of high-throughput sequencing data in taxonomic studies of *Musa* species can provide insights into the relatedness of closely related species as genetic distinction can go as deep as the specimen level. Nevertheless, high-throughput sequencing data have not routinely been incorporated in taxonomic assessments of the *Musa* genus.

The *Musa* genus is known to comprise several taxonomic issues due to the limited availability of high-quality specimens from several parts of these plants [17, 23]. As a consequence, many species are only described based on the morphological characters of one or a few plants, often not seen in the wild but only known from cultivation (e.g. *M*. *haekkinenii* N.S.Lý & Haev.) [26]. To evaluate the taxonomic status of *Musa* species, a thorough study of the diversity within multiple populations of a given (range of) species is necessary in which morphological and molecular characters are jointly assessed. The current study aims to elucidate the evolutionary relationships between two endemic *Musa* species from northern Viet Nam identified as *M*. *viridis* or *M*. *splendida* by using genome-wide genetic polymorphisms called through DArTseq analysis. Based on the findings in this study, we will provide recommendations regarding the taxonomic status of both species.

## Materials & methods

### Taxon sampling and genotyping

**Fig 1:**
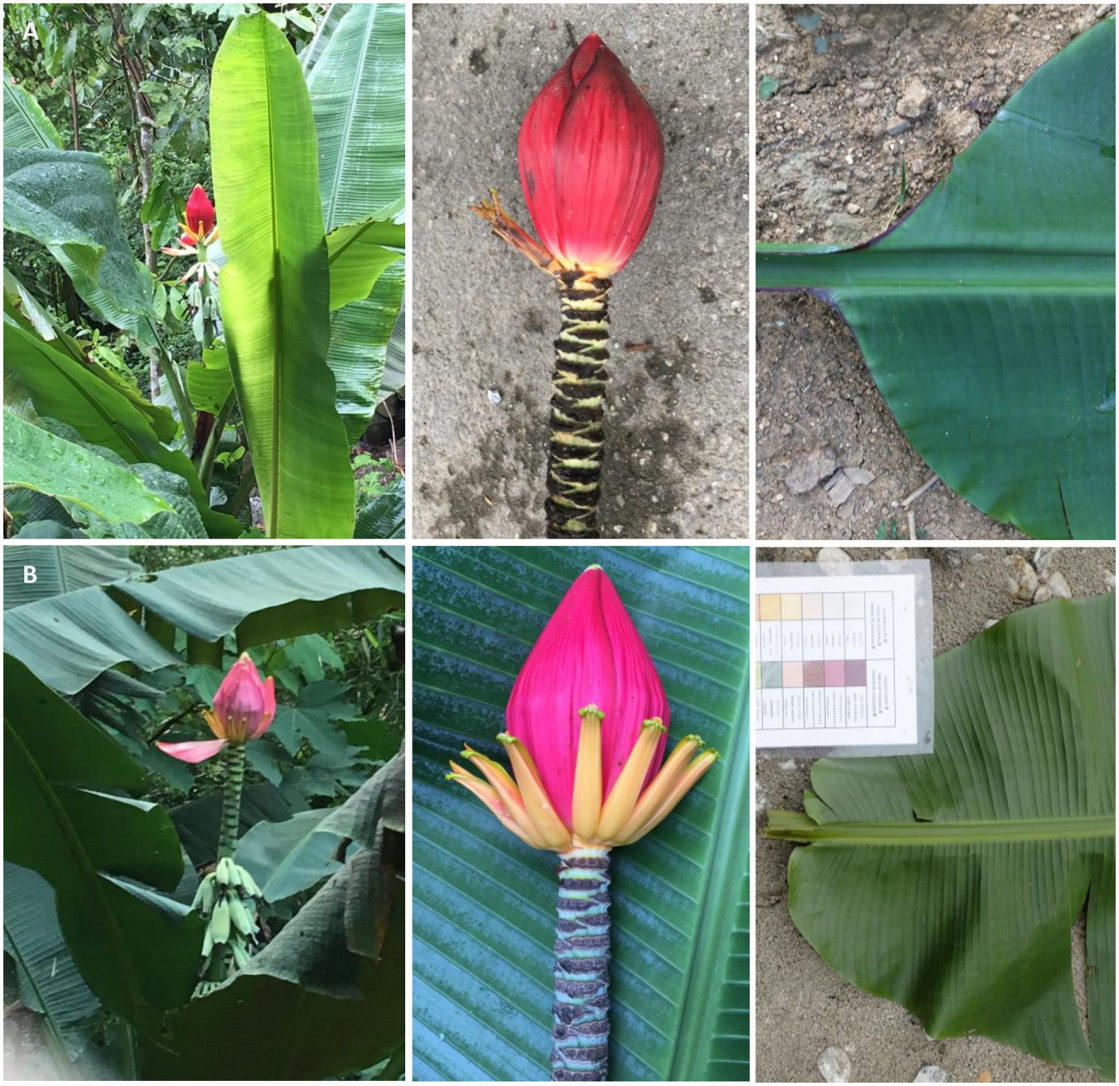
Mature plant (left), male flower bud (middle), and leaf lamina basis (right) of *M*. *splendida* (A) and *M*. *viridis* (B). The difference in flower bract color is used as the main morphological discriminant between these species (red in *M*. *splendida* and pink in *M*. *viridis*).

**Fig 2:**
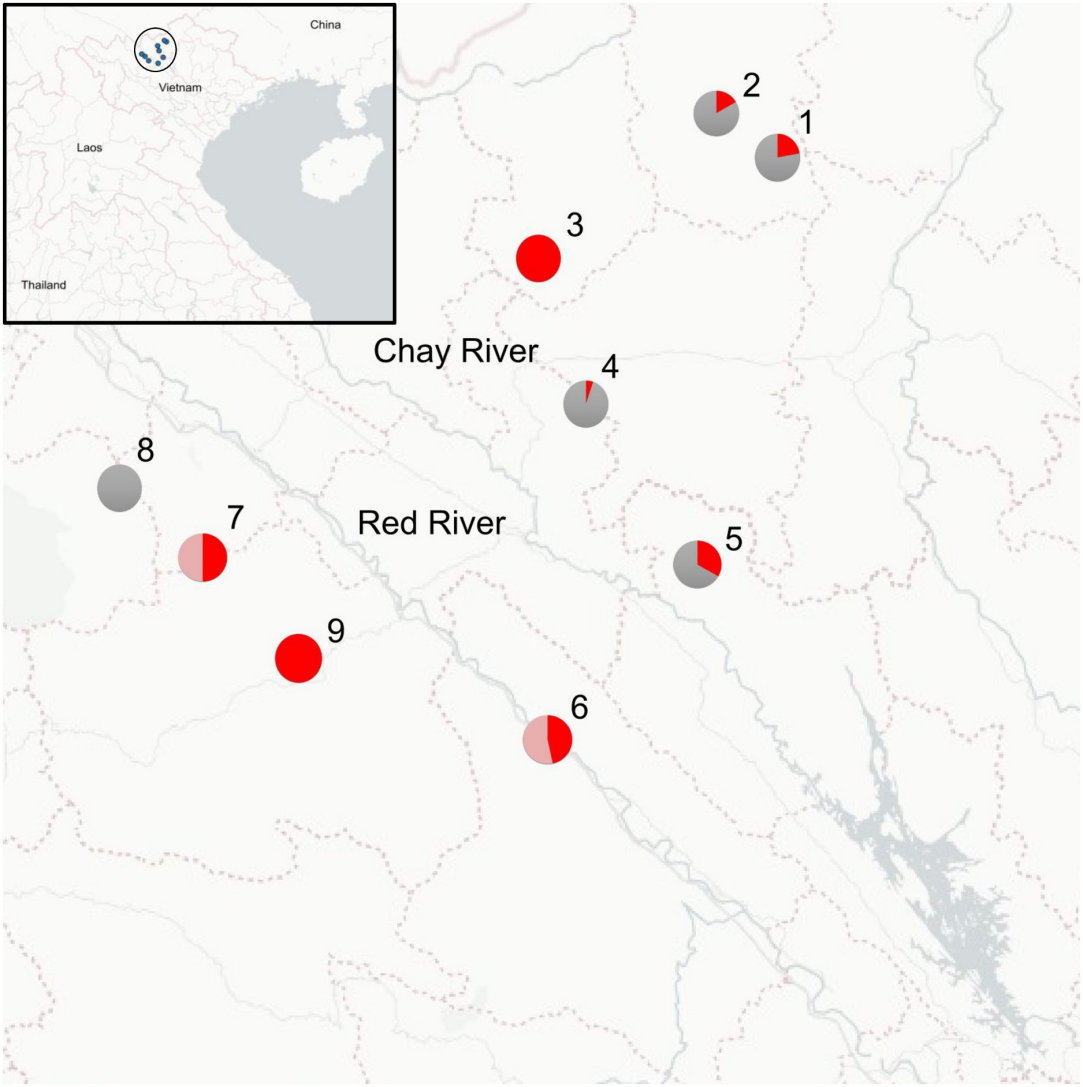
Map of the nine sampling locations of the *M*. *splendida* and *M*. *viridis* accessions in Viet Nam. Labels correspond to the ID of each sampling location and pie charts indicate the proportion of *M*. *splendida* (red), *M*. *viridis* (pink), and unknown individuals (grey) per population. All populations were located close to the Chay river and Red river (blue lines) in the northern part of the country (blue dots in black circle in inset). Map data from © OpenStreetMap under the Open Database Licence [32].

In total, 121 plants (divided over nine populations) were selected for this study. Of these, 53 specimens were morphologically identified as *M*. *splendida*, while 14 specimens were morphologically identified as *M*. *viridis* (Fig 1). The remaining 54 specimens from various populations were only in a vegetative state and thus not identified to species level (further indicated as ‘*Musa* sp.’ throughout the text; S1 Table). Plants were morphologically assigned to a species based on the expertise of local botanists and on characters that were recorded in the field. *Musa* plants used in this study were sampled from nine populations in the Lào Cai, Hà Giang, and Yên Bái provinces in northern Viet Nam (Fig 2), located within the described range of both species [21]. All field work was conducted as part of a Bilateral cooperation project between Meise Botanic Garden (Belgium) and the Plant Resources Center (Ha Noi, Viet Nam), which was permitted by the Vietnamese National Foundation for Science and Technology Development (NAFOSTED). Field permits were obtained for the Yên Bái (564/UBND-NV), Hà Giang (255/SNN-CCLN), and Lào Cai provinces (1060/UBND-NC). A modified cetyltrimethylammonium bromide (CTAB) protocol from [27] was used to extract DNA from 15 mg of silica-dried leaf material per individual. Next, 20 μl of DNA was shipped to Diversity Arrays Technology (DArT, University of Canberra, Bruce, Australia) for an enzyme-based genomic complexity reduction approach with the restriction enzymes PstI and MseI (DArTseq) following [28]. Several molecular studies applied DArTseq on *Musa* plants, showing that it is a reliable method for the identification of genetic variation in these species [29–31]. All DArTseq libraries were subsequently 150 base pairs (bp) single-end sequenced on an Illumina NovaSeq 6000 instrument.

### Read data processing

Demultiplexed reads retrieved from DArT were trimmed to remove barcodes and the PstI restriction site remnant at the 5’-end and the MseI restriction site remnant and adapter sequence at the 3’-end with Cutadapt v3.5 [33] in GBprocesS v4.0.0 [34]. Reads with a barcode shorter than the maximum barcode were 3’ trimmed with Cutadapt to compensate for variation in barcode lengths. In addition, reads shorter than 20 bp were deleted. Afterwards, all reads were filtered for maximum five ambiguous nucleotide calls (Ns) and an average base quality score of 25 using the MaxNFilter and AverageQualityFilter in GBprocesS. Reads with internal intact PstI or MseI restriction sites were discarded as well with the RemovePatternFilter in GBprocesS. All trimmed and quality filtered sequencing data were deposited at the European Nucleotide Archive (ENA) in BioProject PRJEB76413.

Trimmed and filtered reads were mapped onto the *Musa acuminata* subsp. *malaccensis* ‘DH Pahang’ v4 reference genome sequence [35] using the BWA-MEM algorithm in BWA 0.7.17 [36]. This *Musa acuminata* reference genome sequence was the most extensively annotated reference genome sequence at the time of our analysis and was demonstrated to be an effective common reference for closely related *Musa* species [37]. All reads were subsequently indexed, sorted, and filtered for a minimum mapping quality (MAPQ) score of 20 with Samtools v1.17 [38] and tagged with read groups using the Picard AddOrReplaceReadGroups program v3.3.0 [39], resulting in minimum 740,179 and maximum 1,514,724 high-quality mapped reads per sample. Single Nucleotide Polymorphisms (SNPs) in the mapped reads were identified with the Unified Genotyper of the Genome Analysis Toolkit (GATK) v3 [40]. SNP calls were filtered for a minimum minor allele count (MAC) of 4, a minimum SNP quality score of 20, and a minimum genotype quality score of 20 with VCFtools v0.1.17 [41]. Only polymorphic and biallelic SNPs were retained with GATK. Finally, SNP positions with a total read depth below 20, an allele depth below 3, or a data completeness below five percent were removed with a custom python script available on GitLab (https://gitlab.com/ybawin/sequence-data-processing-tetraploids). The data processing work flow and SNP filtering steps were based on approaches applied by previous studies that used similar data [42, 43]. Threshold values for SNP filtering were set after an empirical evaluation of different values on the SNP data of this study.

### Read-backed haplotyping

The start and end position of loci was determined using SMAP *delineate* [44]. The minimum stack depth and cluster depth were set to five and ten, respectively, whereas loci with data in less than five percent of the samples were ignored. Short (< 240 bp) haplotypes were called based on the filtered SNP set and on variation in start and end positions in loci (SMAPs) using SMAP *haplotype-sites* [44]. Haplotypes were called for loci with minimum 20 reads with a minimum MAPQ score of 20 and if they had a minimum frequency of 5 percent in a locus. Haplotypes that only partially aligned to the locus were included following the best practices for GBS data outlined in the SMAP manual (ngs-smap.readthedocs.io), but haplotypes with insertions or deletions on SNP and SMAP positions were removed. Haplotype frequencies were subsequently converted into discrete dominant calls (0 or 1), applying a frequency bound interval of 20 percent.

### Analysis of genetic structure

Based on these haplotype calls, a Jaccard similarity coefficient (J) [45] was calculated for each sample pair and transformed into inversed distances (1–J) using the SMAPapp-Matrix.py script available from the SMAPapps GitLab project (https://gitlab.com/ybawin/smapapps). The resulting genetic distance matrix was used to conduct a Principal Coordinates Analyses (PCoA) with the *cmdscale* function from the stats package in R v4.3.1 [46]. In addition, genetic stratification in our sampling was evaluated based on SNPs in the filtered VCF file using fastStructure v1.0 [47] with all default settings. The number of subpopulations (K) varied between 1 and 9 and the number of subpopulations with the highest likelihood value was 4. Genetic patterns caused by isolation by distance were inferred by a Mantel test using the *mantel*.*rtest* function in the STATS package. The Mantel test was performed on the Jaccard genetic distance matrix and a geographic distance matrix that was created based on longitude and latitude coordinates.

### Phylogenetic tree reconstruction

A nucleotide alignment of consensus sequences was created for each locus delineated with SMAP using the SMAPapp-Alignment.py script in the SMAPapps GitLab project. Consensus sequences consisted of both SNPs and invariable sites, and had less than 25 percent missing nucleotide calls. Only alleles supported by minimum 5 reads and with a minimum frequency of 20 percent in the locus were used for the construction of a consensus sequence. Loci with partially overlapping reads were combined into one locus to reduce redundancy between locus alignments. Consensus sequences with only ambiguous nucleotide calls and invariable sites were discarded as well. A maximum likelihood phylogenetic tree was reconstructed for each locus alignment with IQ-TREE 2 [48] using the ModelFinder tool [49] to select the most appropriate substitution model per locus based on the corrected Akaike Information Criterion (AICc). Branch support values (*i*.*e*. local posterior probability values) were obtained using non-parametric bootstrapping based on 200 bootstrap replicates. Afterwards, all locus consensus trees were combined into one taxon consensus tree with ASTRAL-III [50].

## Results

**Fig 3:**
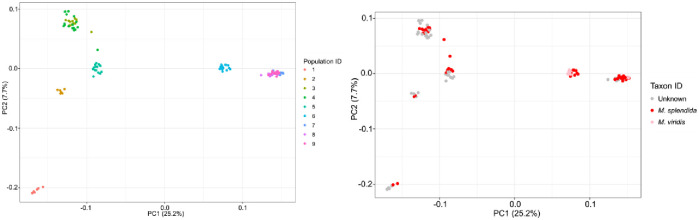
Plot of the first two principal coordinates (PC) that were constructed based on the haplotype variation across the 121 *Musa* individuals. The first and second coordinate explained 25.2% and 7.7% of the total variation in the dataset, respectively. Individuals were colored based on population ID (left) and taxon ID (right).

In total, 50,388 loci were identified in 121 samples, covering 4,629,437 bp (1%) of the *M*. *acuminata* reference genome sequence. After SNP filtering, 51,188 SNPs were retained in all samples. All SNP and SMAP variation was combined into 174,951 haplotypes that were identified in 36,369 loci. The first three principal coordinates jointly explained more than 40% of the total variation in the haplotypes table and partitioned the *Musa* plants into six clusters (Fig 3A). Four out of six clusters consisted exclusively of individuals from one population. The two remaining clusters combined individuals from populations 3 and 4 and from populations 7, 8, and 9, respectively. The first principal coordinate predominantly separated populations 1, 2, 3, 4, and 5 from population 6 and from populations 7, 8, and 9. The second principal coordinate mainly isolated plants from population 1 from all other plants (Fig 3A), while the third principal coordinate confirmed the distinct genetic clustering of populations 5 and 6 into separate groups (S1 Fig). Two clusters consisted of both *M*. *splendida* and *M*. *viridis* plants: the cluster comprising population 6 and the cluster comprising populations 7, 8, and 9 (Fig 3B).

**Fig 4:**
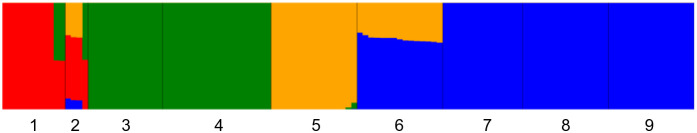
Barplot showing the membership probabilities of each individual to one of the four subpopulations (k = 4). Numbers below the barplot indicate the population ID of the samples.

The number of subpopulations with the highest likelihood value in the fastStructure analysis was four, predominantly corresponding to the following groups: population 1; population 3 and 4; population 5; and population 7, 8, and 9 (Fig 4). Nearly all plants from population 2 were assigned with high membership probabilities to the subpopulation containing population 1 and to the subpopulation containing population 5. These plants also had low membership probabilities to the subpopulation with populations 7, 8, and 9. All individuals from population 6 had substantially high membership probabilities to population 5 and to populations 7, 8, and 9. A few plants from population 1 and 2 were equally assigned to the subpopulation consisting of populations 3 and 4. A positive and significant correlation was found between the genetic distance matrix and the geographic coordinates of the plants based on the results of the Mantel test (observation = 0.79, p-value = 0.0001), providing evidence for a pattern of isolation by distance in the *Musa* populations.

**Fig 5:**
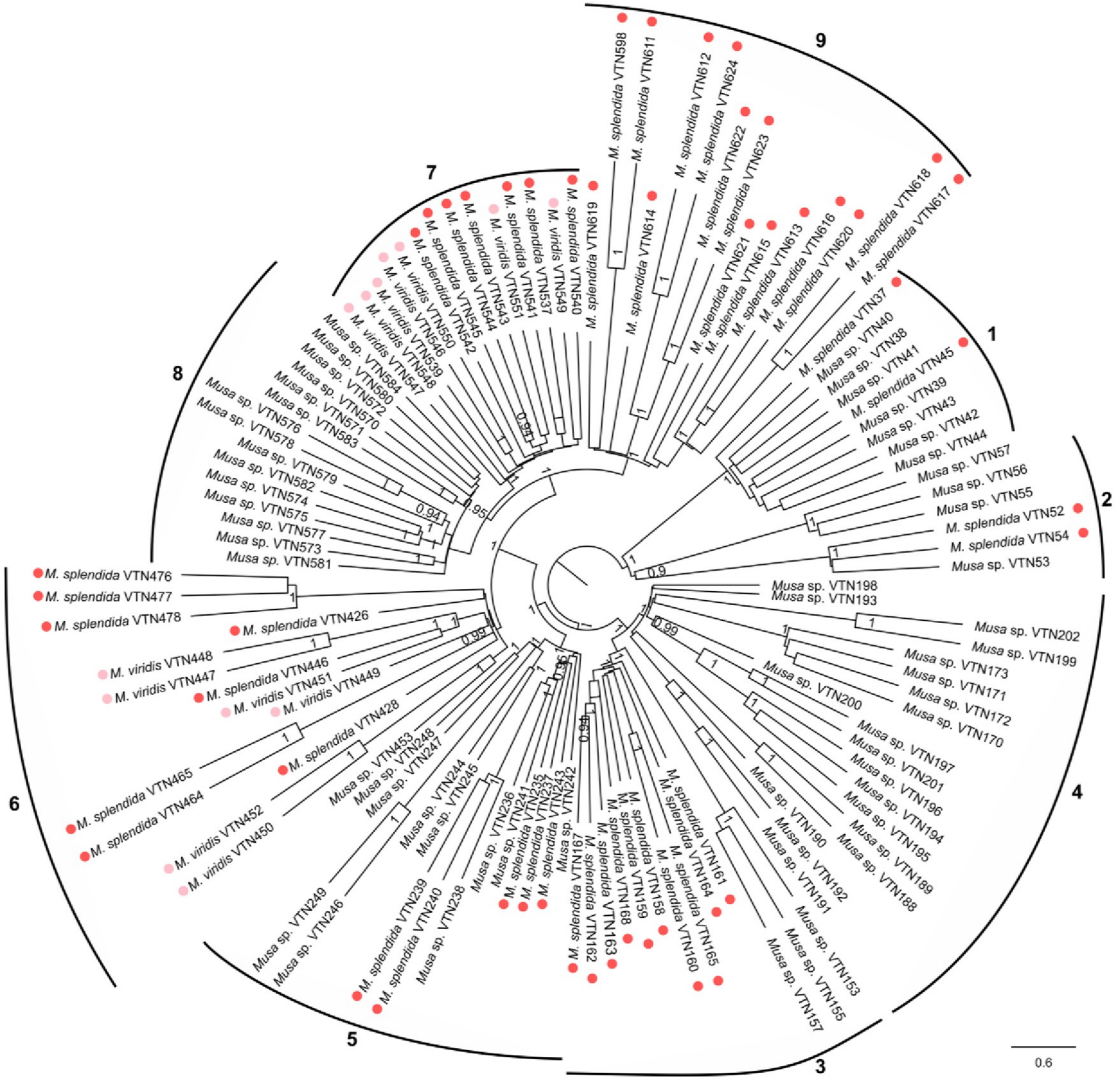
ASTRAL consensus tree of 121 *M*. *splendida* and *M*. *viridis* individuals. All plants were partitioned into nine clades that were indicated by the curved lines and by their population ID next to the tip labels. Node labels are local posterior probability values. Only values of 0.95 or higher are shown.

All locus and SNP data were combined into 11,859 non-overlapping polymorphic locus alignments. The accessions in the ASTRAL consensus tree were subdivided into nine clades, corresponding to their geographic distribution across the study area (Fig 5). Clustering into nine clades was supported by high local posterior probability values (> 0.9). Plants assigned to *M*. *splendida* and *M*. *viridis* clustered together in clades 6 and 7 of the phylogenetic tree.

## Discussion

Recent advances in the acquisition and processing of high-throughput sequencing data enabled the use of genome-wide polymorphic markers to assess genetic relationships in plants. Based on DArTseq data, we studied genetic patterns in plants described as *M*. *splendida* or *M*. *viridis* in northern Viet Nam. The *Musa* individuals clustered based on population origin in either the PCoA plot, fastStructure barplot, and phylogenetic tree, showing a strong isolation-by-distance pattern, rather than based on taxon identity. Neither *Musa splendida* nor *M*. *viridis* were grouped into a separate monophyletic group. Individuals from one species were scattered over multiple clades in the tree, clustering together with other individuals from the same population and not with individuals with the same taxon ID. Consequently, the distinct taxonomic delimitation of *M*. *splendida* and *M*. *viridis* is uncertain. According to their species descriptions, the main difference between these two taxa is the color of the bracts of the male flower bud that ranges from red and (pinkish)-red (*M*. *splendida*) to pink (*M*. *viridis*) [22–24]. Variation in male bract color of banana plants is often caused by differences in the composition of anthocyanins [51, 52]. Although the proportion of different types of anthocyanins in bracts may vary across individuals of the same species, their resulting coloration patterns were so far always considered to be relatively stable within one species [51, 53]. Therefore, the color of male flower bracts was assumed to be a character with high taxonomic value in Musaceae [51]. Nevertheless, variation in flower bract color has also been observed in *M*. *acuminata* subsp. *banksii* (F.Muell.) N.W.Simmonds, demonstrating that color variation in flower bracts can even occur within a *Musa* subspecies [54]. Flower color is determined by a variety of abiotic and biotic factors including pollinator diversity, solar radiation, and mean annual rainfall [55]. Different environmental conditions may slightly alter the color of flower bracts, resulting in intraspecific variation. For example, a study in the *Epimedium* L. genus (Berberidaceae) found large intraspecific variation in flower color, showing that flower color is an unsuitable character for species delimitation in this plant genus [56]. Analogously, the variation in the color of male flower bracts is not a stable character to classify plants assigned to *M*. *splendida* and *M*. *viridis* into two distinct species. Our field observations of the shape of the leaf lamina basis of *Musa* plants showed that these characters are also not stable across individuals assigned to the same species. In particular, the shape of the leaf lamina basis varied between inner and other vegetative leaves and was often deformed due to mechanical damage. This genetic study provides evidence that supports the aggregation of all plants in our sampling into one species named *M*. *splendida*. This name has priority over *M*. *viridis* because it is the oldest available name (ICN Art. 11.3) [57].

The output of the PCoA and fastStructure analysis showed that the *Musa* populations are aggregated into larger genetic groups. This subdivision predominantly corresponded to their geographic distribution across the study area and the topological variation in the landscape created by the courses of the Chay River and Red River. Subpopulations 1, 2, and 3 in the fastStructure analyses were sampled at a considerable distance from each other in an area east of the Chay River, whereas subpopulation 4 was located east of the Red River. A pronounced population structure was also found in *M*. *balbisiana* and *M*. *itinerans* Cheesman populations from Vietnam, Laos, and China, which could also be linked to geographic variation across their distribution area caused by river basins and mountain ranges [18, 37, 58]. Moreover, similar patterns of genetic differentiation in *Zingiber corallinum* Hance populations (Zingiberaceae) from Hainan were attributed to their isolated position on mountain ranges separated by the Changhua river valley [59]. Consequently, river systems and their associated topological variation in northern Viet Nam and neighboring regions seem to form an effective seed and pollen dispersal barrier for several Zingiberales species. However, other environmental factors such as climate variation may additionally determine population genetic structure in *Musa* species, as shown for *Zingiber nudicarpum* D.Fang [59]. The *Musa* populations in our study displayed a strong pattern of isolation by distance. Such patterns were not observed for Vietnamese *M*. *balbisiana* populations, which was attributed to human translocations of *M*. *balbisiana* plants for nutritional purposes or fiber production [18, 37]. Although *M*. *splendida* and *M*. *viridis* are used as ornamental species and as a flavor enhancer of rice wine, human translocations may not substantially have changed isolation-by-distance patterns in these taxa. Most populations in our sampling were not admixed, corroborating the limited degree of admixture that was also found in *M*. *balbisiana* populations sampled from the same geographic area [18]. A study of Vietnamese ginseng populations (*Panax vietnamensis* Ha & Grushv.) found evidence for much higher levels of admixture, suggesting that admixture levels vary among different plant groups in Viet Nam [60]. Only populations 2 and 6 displayed considerable evidence for admixture between two genetic groups across all individuals within these populations. The intermediate geographic location of population 6 between subpopulations 3 and 4 may support the possibility of an admixed origin of this population, although these patterns can also be explained by isolation-by-distance [61] Important to note is that we did not find a significant signal of hybridization among the different lineages in our sampling. Hence, these patterns of admixture could not directly be linked to the *M*. *splendida* or *M*. *viridis* individuals.

Given the high morphological resemblance between *M*. *splendida* and other *Musa* species, a more elaborate study of the genetic relationships between *M*. *splendida* and closely related congeneric species may clarify their taxonomic status. As several *Musa* species were described based on one or a few herbarium specimens, the intraspecific variation in those species has often been ignored. For instance, the species *M*. *exotica* R.V.Valmayor is only found in the Cuc Phuong Forest Reservation in the Ninh Binh province in Viet Nam, where it was assumedly planted as an ornamental [21, 23]. As no wild populations of this species are currently known, its conservation status is unclear. It differs morphologically from *M*. *viridis* by its orange-red bracts and yellow fruits [23]. Nevertheless, as the color of male flower bracts is seemingly not a stable morphological character of the *Musa* species analyzed in this study, the value of this character to discriminate between other *Musa* species is questionable. A thorough assessment of all *Callimusa* species in southern China and northern Viet Nam based on multiple individuals per species and on a combination of morphological and genetic data is necessary to correctly identify the species relationships within this group of banana species.

## Conclusions

Plants identified as *M*. *viridis* or *M*. *splendida* from northern Viet Nam are genetically more related to plants from the same population than to plants with the same taxon identity. Consequently, we propose to describe all plants assigned to these two taxa in Viet Nam as *M*. *splendida*. The results of this study show that the color of male flower bracts may not always be a reliable character for the delimitation of species in the *Musa* genus, advocating for genetic assessments of other species groups within this genus that mainly differ based on variation within a single character. Taxonomic and molecular research on wild *Musa* species may benefit from the development of an accessible *ex situ* living collection of well-documented reference plants that are morphologically and genetically completely characterized. Such efforts may overcome the lack of high-quality herbarium specimens of *Musa* species. Furthermore, given the evidence for genetic structure and isolation-by-distance in wild *Musa* populations, future studies may focus on the inference of correlations between genetic patterns and environmental factors to clarify which factors contribute to the genetic structure in *Musa* species. In addition, research efforts might be more directed towards the study of gene flow within and between *Musa* species, disentangling the effects of admixture and hybridization from isolation-by-distance patterns. Such insights would be valuable for the *in-situ* conservation of these banana genetic resources.

### Taxonomic treatment

***Musa splendida*** A.Chev., Rev. Bot. Appl. Agric. Trop. 14: 517. 1934. emend. N.S.Lý et al. (Lý & al. 2018: 285)–Lectotype: VIET NAM. Tonkin: Laokay province, Muong-Xen, 4 Dec 1913, *A*. *Chevalier s*.*n*. (P barcode P01767056!).–Epitype (designated by Lý et al. in Phytotaxa 351: 285. 2018):–VIET NAM. Haut-Tonkin: Laokay Province, Phu Lu, 6 Dec 1935, *M*. *Poilane 24969* (P barcodes P00742068!, P00742069! [mounted on two sheets]).

= *Musa viridis* R.V.Valmayor, Đ.D.Lê & Häkkinen, Philipp. Agric. Sci. 87(1): 115. 2004–Holotype: VIET NAM. Van Chan: Yen Bai province, 29 Nov 1994, *L*. *D*. *Danh*, *1–052* (PHH barcode PHH002), **syn. nov.**

### Emended description

The description is the same as [23], but emended as follows: leaf basis rounded to cuneate on both sides; male bract orange-red to bright red or pink-lilac on both surfaces, fading to yellow towards the base.

## Supporting information

S1 Fig Plots of the second and third principal coordinates (PC) that were constructed based on the haplotype variation across the 121 *Musa* individuals. The second and third coordinate explained 7.7% and 7.6% of the total variation in the dataset, respectively. Individuals were colored based on population ID (upper plot) and taxon ID (lower plot).
(PDF)

S1 Table Overview of the *Musa* plants included this study. Collection ID, taxon ID, country, district, and locality of origin, longitude and latitude coordinates, population ID, and the color of their male flower bracts are listed.
(DOCX)
