# Supplementary material for: A splendid banana enigma: Phylogenomic assessment of Vietnamese Musa splendida and Musa viridis populations shows that they are conspecific
Source: PLoS One. 2025 Feb 11;20(2):e0318252. doi: 10.1371/journal.pone.0318252 (PMC11813090; doi:10.1371/journal.pone.0318252)
Supplement: S1 Fig — The second and third coordinate explained 7.7% and 7.6% of the total variation in the dataset, respectively. Individuals were colored based on population ID (upper plot) and taxon ID (lower plot). (PDF) [file pone.0318252.s001.pdf]

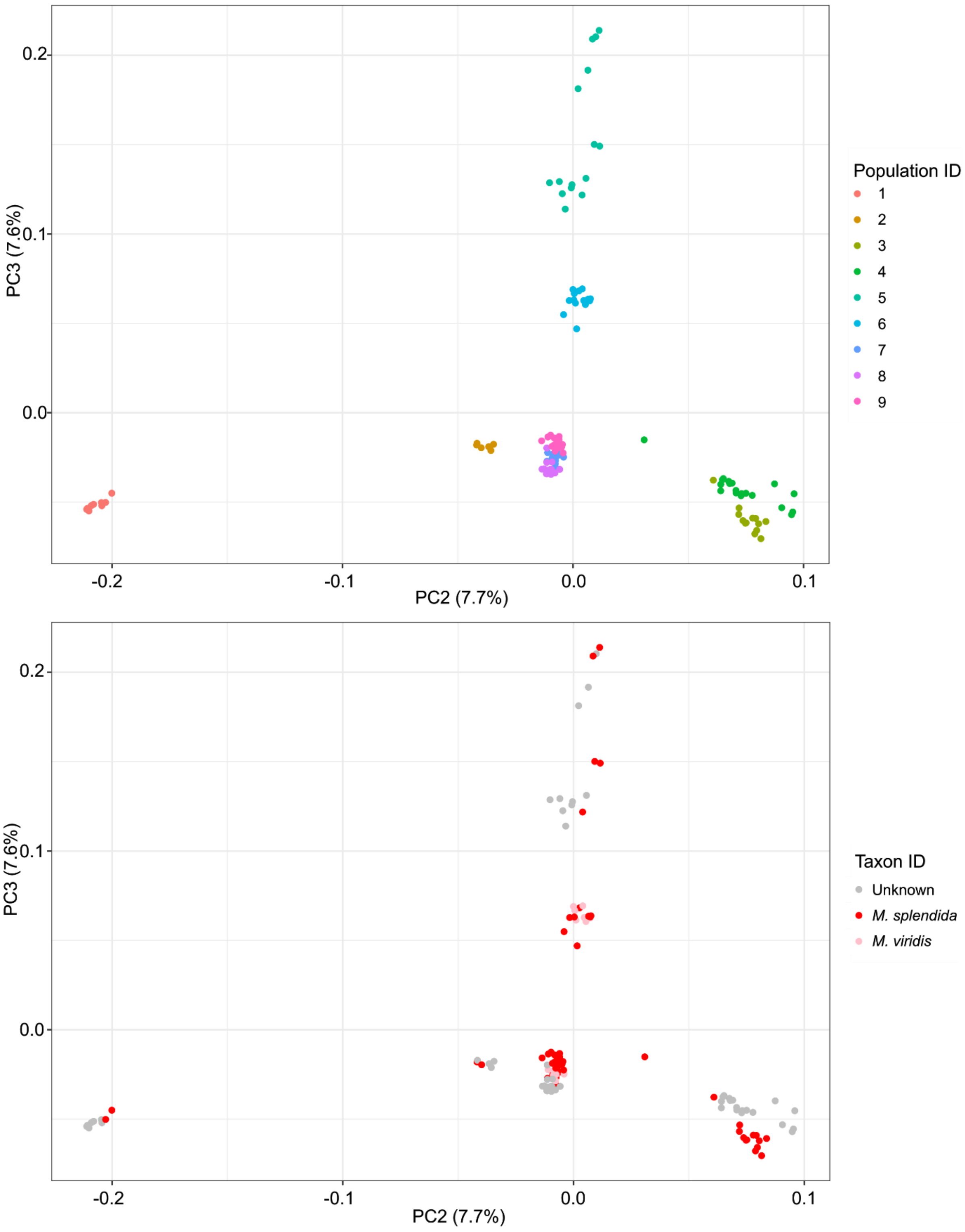

Fig. S1. Plots of the second and third principal coordinates (PC) that were constructed based on the haplotype variation across the 121 *Musa* individuals. The second and third coordinate explained 7.7% and 7.6% of the total variation in the dataset, respectively. Individuals were colored based on population ID (upper plot) and taxon ID (lower plot).
