# Supplementary material for: A splendid banana enigma: Phylogenomic assessment of Vietnamese Musa splendida and Musa viridis populations shows that they are conspecific
Source: PLoS One. 2025 Feb 11;20(2):e0318252. doi: 10.1371/journal.pone.0318252 (PMC11813090; doi:10.1371/journal.pone.0318252)
Supplement: S1 Table — Collection ID, taxon ID, country, district, and locality of origin, longitude and latitude coordinates, population ID, and the color of their male flower bracts are listed. (DOCX) [file pone.0318252.s002.docx]

| **Table S1.** Overview of the *Musa* plants included this study showing their collection ID, taxon ID, country, district, and locality of origin, longitude and latitude coordinates, population ID, and the color of their male flower bracts. | | | | | | | | | | |
| --- | --- | --- | --- | --- | --- | --- | --- | --- | --- | --- |
| **Collection ID** | **Genus** | **Species** | **Collection date** | **Country** | **District** | **Locality** | **Latitude** | **Longitude** | **Population ID** | **Color male flower bracts** |
| VTN37 | *Musa* | *splendida* | 16/04/2018 | Vietnam | Huang Su Phi | Nam Ty | 22.63047 | 104.7568 | 1 | Red |
| VTN38 | *Musa* | sp. | 16/04/2018 | Vietnam | Huang Su Phi | Nam Ty | 22.63047 | 104.7568 | 1 | unknown |
| VTN39 | *Musa* | sp. | 16/04/2018 | Vietnam | Huang Su Phi | Nam Ty | 22.63047 | 104.7568 | 1 | unknown |
| VTN40 | *Musa* | sp. | 16/04/2018 | Vietnam | Huang Su Phi | Nam Ty | 22.63047 | 104.7568 | 1 | unknown |
| VTN41 | *Musa* | sp. | 16/04/2018 | Vietnam | Huang Su Phi | Nam Ty | 22.63047 | 104.7568 | 1 | unknown |
| VTN42 | *Musa* | sp. | 16/04/2018 | Vietnam | Huang Su Phi | Nam Ty | 22.63047 | 104.7568 | 1 | unknown |
| VTN43 | *Musa* | sp. | 16/04/2018 | Vietnam | Huang Su Phi | Nam Ty | 22.63047 | 104.7568 | 1 | unknown |
| VTN44 | *Musa* | sp. | 16/04/2018 | Vietnam | Huang Su Phi | Nam Ty | 22.63047 | 104.7568 | 1 | unknown |
| VTN45 | *Musa* | *splendida* | 16/04/2018 | Vietnam | Huang Su Phi | Nam Ty | 22.63047 | 104.7568 | 1 | Red |
| VTN52 | *Musa* | *splendida* | 17/04/2018 | Vietnam | Huang Su Phi | Ban Luoc | 22.66664 | 104.697 | 2 | unknown |
| VTN53 | *Musa* | sp. | 17/04/2018 | Vietnam | Huang Su Phi | Ban Luoc | 22.66664 | 104.697 | 2 | unknown |
| VTN54 | *Musa* | *splendida* | 17/04/2018 | Vietnam | Huang Su Phi | Ban Luoc | 22.66664 | 104.697 | 2 | Red |
| VTN55 | *Musa* | sp. | 17/04/2018 | Vietnam | Huang Su Phi | Ban Luoc | 22.66664 | 104.697 | 2 | unknown |
| VTN56 | *Musa* | sp. | 17/04/2018 | Vietnam | Huang Su Phi | Ban Luoc | 22.66664 | 104.697 | 2 | unknown |
| VTN57 | *Musa* | sp. | 17/04/2018 | Vietnam | Huang Su Phi | Ban Luoc | 22.66664 | 104.697 | 2 | unknown |
| VTN153 | *Musa* | *splendida* | 18/04/2018 | Vietnam | Xim Man | Na Chi | 22.52097 | 104.5043 | 3 | Pinkish-red |
| VTN155 | *Musa* | *splendida* | 18/04/2018 | Vietnam | Xim Man | Na Chi | 22.52097 | 104.5043 | 3 | Pinkish-red |
| VTN157 | *Musa* | *splendida* | 18/04/2018 | Vietnam | Xim Man | Na Chi | 22.52097 | 104.5043 | 3 | Pinkish-red |
| VTN158 | *Musa* | *splendida* | 18/04/2018 | Vietnam | Xim Man | Na Chi | 22.52097 | 104.5043 | 3 | Red |
| VTN159 | *Musa* | *splendida* | 18/04/2018 | Vietnam | Xim Man | Na Chi | 22.52097 | 104.5043 | 3 | Red |
| VTN160 | *Musa* | *splendida* | 18/04/2018 | Vietnam | Xim Man | Na Chi | 22.52097 | 104.5043 | 3 | Red |
| VTN161 | *Musa* | *splendida* | 18/04/2018 | Vietnam | Xim Man | Na Chi | 22.52097 | 104.5043 | 3 | Red |
| VTN162 | *Musa* | *splendida* | 18/04/2018 | Vietnam | Xim Man | Na Chi | 22.52097 | 104.5043 | 3 | Red |
| VTN163 | *Musa* | *splendida* | 18/04/2018 | Vietnam | Xim Man | Na Chi | 22.52097 | 104.5043 | 3 | Red |
| VTN164 | *Musa* | *splendida* | 18/04/2018 | Vietnam | Xim Man | Na Chi | 22.52097 | 104.5043 | 3 | Red |
| VTN165 | *Musa* | *splendida* | 18/04/2018 | Vietnam | Xim Man | Na Chi | 22.52097 | 104.5043 | 3 | Red |
| VTN167 | *Musa* | *splendida* | 18/04/2018 | Vietnam | Xim Man | Na Chi | 22.52097 | 104.5043 | 3 | Red |
| VTN168 | *Musa* | *splendida* | 18/04/2018 | Vietnam | Xim Man | Na Chi | 22.52097 | 104.5043 | 3 | Red |
| VTN170 | *Musa* | *splendida* | 19/04/2018 | Vietnam | Quang Binh | Yin Thanh | 22.38578 | 104.5425 | 4 | Pinkish-red |
| VTN171 | *Musa* | sp. | 19/04/2018 | Vietnam | Quang Binh | Yin Thanh | 22.38578 | 104.5425 | 4 | unknown |
| VTN172 | *Musa* | sp. | 19/04/2018 | Vietnam | Quang Binh | Yin Thanh | 22.38578 | 104.5425 | 4 | unknown |
| VTN173 | *Musa* | sp. | 19/04/2018 | Vietnam | Quang Binh | Yin Thanh | 22.38578 | 104.5425 | 4 | unknown |
| VTN188 | *Musa* | sp. | 19/04/2018 | Vietnam | Quang Binh | Yin Thanh | 22.38578 | 104.5425 | 4 | unknown |
| VTN189 | *Musa* | sp. | 19/04/2018 | Vietnam | Quang Binh | Yin Thanh | 22.38578 | 104.5425 | 4 | unknown |
| VTN190 | *Musa* | sp. | 19/04/2018 | Vietnam | Quang Binh | Yin Thanh | 22.38578 | 104.5425 | 4 | unknown |
| VTN191 | *Musa* | sp. | 19/04/2018 | Vietnam | Quang Binh | Yin Thanh | 22.38578 | 104.5425 | 4 | unknown |
| VTN192 | *Musa* | sp. | 19/04/2018 | Vietnam | Quang Binh | Yin Thanh | 22.38578 | 104.5425 | 4 | unknown |
| VTN193 | *Musa* | sp. | 19/04/2018 | Vietnam | Quang Binh | Yin Thanh | 22.38867 | 104.5432 | 4 | unknown |
| VTN194 | *Musa* | sp. | 19/04/2018 | Vietnam | Quang Binh | Yin Thanh | 22.39397 | 104.5444 | 4 | unknown |
| VTN195 | *Musa* | sp. | 19/04/2018 | Vietnam | Quang Binh | Yin Thanh | 22.39397 | 104.5444 | 4 | unknown |
| VTN196 | *Musa* | sp. | 19/04/2018 | Vietnam | Quang Binh | Yin Thanh | 22.39397 | 104.5444 | 4 | unknown |
| VTN197 | *Musa* | sp. | 19/04/2018 | Vietnam | Quang Binh | Yin Thanh | 22.39397 | 104.5444 | 4 | unknown |
| VTN201 | *Musa* | sp. | 19/04/2018 | Vietnam | Quang Binh | Yin Thanh | 22.39397 | 104.5444 | 4 | unknown |
| VTN198 | *Musa* | sp. | 19/04/2018 | Vietnam | Quang Binh | Yin Thanh | 22.39397 | 104.5444 | 4 | unknown |
| VTN199 | *Musa* | sp. | 19/04/2018 | Vietnam | Quang Binh | Yin Thanh | 22.39397 | 104.5444 | 4 | unknown |
| VTN200 | *Musa* | sp. | 19/04/2018 | Vietnam | Quang Binh | Yin Thanh | 22.39397 | 104.5444 | 4 | unknown |
| VTN202 | *Musa* | sp. | 19/04/2018 | Vietnam | Quang Binh | Yin Thanh | 22.39397 | 104.5444 | 4 | unknown |
| VTN235 | *Musa* | *splendida* | 20/04/2018 | Vietnam | Luc Yen | Lam Thuong | 22.20628 | 104.6623 | 5 | Pinkish-red |
| VTN236 | *Musa* | sp. | 20/04/2018 | Vietnam | Luc Yen | Lam Thuong | 22.20628 | 104.6623 | 5 | unknown |
| VTN237 | *Musa* | *splendida* | 20/04/2018 | Vietnam | Luc Yen | Lam Thuong | 22.20628 | 104.6623 | 5 | Pinkish-red |
| VTN238 | *Musa* | sp. | 20/04/2018 | Vietnam | Luc Yen | Lam Thuong | 22.20628 | 104.6623 | 5 | unknown |
| VTN239 | *Musa* | *splendida* | 20/04/2018 | Vietnam | Luc Yen | Lam Thuong | 22.20628 | 104.6623 | 5 | Pinkish-red |
| VTN240 | *Musa* | *splendida* | 20/04/2018 | Vietnam | Luc Yen | Lam Thuong | 22.20628 | 104.6623 | 5 | Pinkish-red |
| VTN241 | *Musa* | sp. | 20/04/2018 | Vietnam | Luc Yen | Lam Thuong | 22.20628 | 104.6623 | 5 | unknown |
| VTN242 | *Musa* | sp. | 20/04/2018 | Vietnam | Luc Yen | Lam Thuong | 22.20628 | 104.6623 | 5 | unknown |
| VTN243 | *Musa* | *splendida* | 20/04/2018 | Vietnam | Luc Yen | Lam Thuong | 22.20628 | 104.6623 | 5 | pinkish-red |
| VTN244 | *Musa* | sp. | 20/04/2018 | Vietnam | Luc Yen | Lam Thuong | 22.20703 | 104.6703 | 5 | unknown |
| VTN245 | *Musa* | sp. | 20/04/2018 | Vietnam | Luc Yen | Lam Thuong | 22.20703 | 104.6703 | 5 | unknown |
| VTN246 | *Musa* | sp. | 20/04/2018 | Vietnam | Luc Yen | Lam Thuong | 22.20703 | 104.6703 | 5 | unknown |
| VTN247 | *Musa* | sp. | 20/04/2018 | Vietnam | Luc Yen | Lam Thuong | 22.20703 | 104.6703 | 5 | unknown |
| VTN248 | *Musa* | sp. | 20/04/2018 | Vietnam | Luc Yen | Lam Thuong | 22.20703 | 104.6703 | 5 | unknown |
| VTN249 | *Musa* | sp. | 20/04/2018 | Vietnam | Luc Yen | Lam Thuong | 22.20703 | 104.6703 | 5 | unknown |
| VTN426 | *Musa* | *splendida* | 23/04/2018 | Vietnam | Van Yen | Lam Giang | 22.04311 | 104.5145 | 6 | Red |
| VTN428 | *Musa* | *splendida* | 23/04/2018 | Vietnam | Van Yen | Lam Giang | 22.04311 | 104.5145 | 6 | Red |
| VTN446 | *Musa* | *splendida* | 23/04/2018 | Vietnam | Van Yen | Lam Giang | 22.04533 | 104.5103 | 6 | Red |
| VTN447 | *Musa* | *viridis* | 23/04/2018 | Vietnam | Van Yen | Lam Giang | 22.04533 | 104.5103 | 6 | Pink |
| VTN448 | *Musa* | *viridis* | 23/04/2018 | Vietnam | Van Yen | Lam Giang | 22.04533 | 104.5103 | 6 | Pink |
| VTN449 | *Musa* | *viridis* | 23/04/2018 | Vietnam | Van Yen | Lam Giang | 22.04533 | 104.5103 | 6 | Pink |
| VTN450 | *Musa* | *viridis* | 23/04/2018 | Vietnam | Van Yen | Lam Giang | 22.04533 | 104.5103 | 6 | Pink |
| VTN451 | *Musa* | *viridis* | 23/04/2018 | Vietnam | Van Yen | Lam Giang | 22.04533 | 104.5103 | 6 | Pink |
| VTN452 | *Musa* | *viridis* | 23/04/2018 | Vietnam | Van Yen | Lam Giang | 22.04533 | 104.5103 | 6 | Pink |
| VTN453 | *Musa* | *viridis* | 23/04/2018 | Vietnam | Van Yen | Lam Giang | 22.04533 | 104.5103 | 6 | Pink |
| VTN464 | *Musa* | *splendida* | 23/04/2018 | Vietnam | Van Yen | Lam Giang | 22.045 | 104.5089 | 6 | Red |
| VTN465 | *Musa* | *splendida* | 23/04/2018 | Vietnam | Van Yen | Lam Giang | 22.045 | 104.5089 | 6 | Red |
| VTN476 | *Musa* | *splendida* | 23/04/2018 | Vietnam | Van Yen | Lam Giang | 22.04447 | 104.5074 | 6 | Red |
| VTN477 | *Musa* | *splendida* | 23/04/2018 | Vietnam | Van Yen | Lam Giang | 22.04447 | 104.5074 | 6 | Red |
| VTN478 | *Musa* | *splendida* | 23/04/2018 | Vietnam | Van Yen | Lam Giang | 22.04447 | 104.5074 | 6 | Red |
| VTN537 | *Musa* | *splendida* | 26/04/2018 | Vietnam | Bao Thang | Phu Nhuan | 22.23772 | 104.1374 | 7 | Red |
| VTN539 | *Musa* | *splendida* | 26/04/2018 | Vietnam | Bao Thang | Phu Nhuan | 22.23772 | 104.1374 | 7 | Red |
| VTN540 | *Musa* | *splendida* | 26/04/2018 | Vietnam | Bao Thang | Phu Nhuan | 22.23772 | 104.1374 | 7 | Red |
| VTN541 | *Musa* | *splendida* | 26/04/2018 | Vietnam | Bao Thang | Phu Nhuan | 22.23772 | 104.1374 | 7 | Red |
| VTN542 | *Musa* | *splendida* | 26/04/2018 | Vietnam | Bao Thang | Phu Nhuan | 22.23772 | 104.1374 | 7 | Red |
| VTN543 | *Musa* | *splendida* | 26/04/2018 | Vietnam | Bao Thang | Phu Nhuan | 22.23772 | 104.1374 | 7 | Red |
| VTN544 | *Musa* | *splendida* | 26/04/2018 | Vietnam | Bao Thang | Phu Nhuan | 22.23772 | 104.1374 | 7 | Red |
| VTN545 | *Musa* | *viridis* | 26/04/2018 | Vietnam | Bao Thang | Phu Nhuan | 22.23772 | 104.1374 | 7 | Pink |
| VTN546 | *Musa* | *viridis* | 26/04/2018 | Vietnam | Bao Thang | Phu Nhuan | 22.23772 | 104.1374 | 7 | Pink |
| VTN547 | *Musa* | *viridis* | 26/04/2018 | Vietnam | Bao Thang | Phu Nhuan | 22.23772 | 104.1374 | 7 | Pink |
| VTN548 | *Musa* | *viridis* | 26/04/2018 | Vietnam | Bao Thang | Phu Nhuan | 22.23772 | 104.1374 | 7 | Pink |
| VTN549 | *Musa* | *viridis* | 26/04/2018 | Vietnam | Bao Thang | Phu Nhuan | 22.23772 | 104.1374 | 7 | Pink |
| VTN550 | *Musa* | *viridis* | 26/04/2018 | Vietnam | Bao Thang | Phu Nhuan | 22.23772 | 104.1374 | 7 | Pink |
| VTN551 | *Musa* | *viridis* | 26/04/2018 | Vietnam | Bao Thang | Phu Nhuan | 22.23772 | 104.1374 | 7 | Pink |
| VTN570 | *Musa* | sp. | 27/04/2018 | Vietnam | Bao Thang | Suoi Thau | 22.298 | 104.0424 | 8 | Red or pink |
| VTN571 | *Musa* | sp. | 27/04/2018 | Vietnam | Bao Thang | Suoi Thau | 22.298 | 104.0424 | 8 | Red or pink |
| VTN572 | *Musa* | sp. | 27/04/2018 | Vietnam | Bao Thang | Suoi Thau | 22.298 | 104.0424 | 8 | Red or pink |
| VTN573 | *Musa* | sp. | 27/04/2018 | Vietnam | Bao Thang | Suoi Thau | 22.298 | 104.0424 | 8 | Red or pink |
| VTN574 | *Musa* | sp. | 27/04/2018 | Vietnam | Bao Thang | Suoi Thau | 22.298 | 104.0424 | 8 | Red or pink |
| VTN575 | *Musa* | sp. | 27/04/2018 | Vietnam | Bao Thang | Suoi Thau | 22.298 | 104.0424 | 8 | Red or pink |
| VTN576 | *Musa* | sp. | 27/04/2018 | Vietnam | Bao Thang | Suoi Thau | 22.298 | 104.0424 | 8 | Red or pink |
| VTN577 | *Musa* | sp. | 27/04/2018 | Vietnam | Bao Thang | Suoi Thau | 22.298 | 104.0424 | 8 | Red or pink |
| VTN578 | *Musa* | sp. | 27/04/2018 | Vietnam | Bao Thang | Suoi Thau | 22.298 | 104.0424 | 8 | Red or pink |
| VTN579 | *Musa* | sp. | 27/04/2018 | Vietnam | Bao Thang | Suoi Thau | 22.298 | 104.0424 | 8 | Red or pink |
| VTN580 | *Musa* | sp. | 27/04/2018 | Vietnam | Bao Thang | Suoi Thau | 22.298 | 104.0424 | 8 | Red or pink |
| VTN581 | *Musa* | sp. | 27/04/2018 | Vietnam | Bao Thang | Suoi Thau | 22.298 | 104.0424 | 8 | Red or pink |
| VTN582 | *Musa* | sp. | 27/04/2018 | Vietnam | Bao Thang | Suoi Thau | 22.298 | 104.0424 | 8 | Red or pink |
| VTN583 | *Musa* | sp. | 27/04/2018 | Vietnam | Bao Thang | Suoi Thau | 22.298 | 104.0424 | 8 | Red or pink |
| VTN584 | *Musa* | sp. | 27/04/2018 | Vietnam | Bao Thang | Suoi Thau | 22.298 | 104.0424 | 8 | Red or pink |
| VTN598 | *Musa* | *splendida* | 28/04/2018 | Vietnam | Van Ban | Khanh Yen Thuong | 22.11497 | 104.2439 | 9 | Red |
| VTN611 | *Musa* | *splendida* | 28/04/2018 | Vietnam | Van Ban | Khanh Yen Thuong | 22.11497 | 104.2439 | 9 | Red |
| VTN612 | *Musa* | *splendida* | 28/04/2018 | Vietnam | Van Ban | Khanh Yen Thuong | 22.11497 | 104.2439 | 9 | Red |
| VTN613 | *Musa* | *splendida* | 28/04/2018 | Vietnam | Van Ban | Khanh Yen Thuong | 22.11497 | 104.2439 | 9 | Red |
| VTN614 | *Musa* | *splendida* | 28/04/2018 | Vietnam | Van Ban | Khanh Yen Thuong | 22.11497 | 104.2439 | 9 | Red |
| VTN615 | *Musa* | *splendida* | 28/04/2018 | Vietnam | Van Ban | Khanh Yen Thuong | 22.11497 | 104.2439 | 9 | Red |
| VTN616 | *Musa* | *splendida* | 28/04/2018 | Vietnam | Van Ban | Khanh Yen Thuong | 22.11497 | 104.2439 | 9 | Red |
| VTN617 | *Musa* | *splendida* | 28/04/2018 | Vietnam | Van Ban | Khanh Yen Thuong | 22.11497 | 104.2439 | 9 | Red |
| VTN618 | *Musa* | *splendida* | 28/04/2018 | Vietnam | Van Ban | Khanh Yen Thuong | 22.11497 | 104.2439 | 9 | Red |
| VTN619 | *Musa* | *splendida* | 28/04/2018 | Vietnam | Van Ban | Khanh Yen Thuong | 22.11497 | 104.2439 | 9 | Red |
| VTN620 | *Musa* | *splendida* | 28/04/2018 | Vietnam | Van Ban | Khanh Yen Thuong | 22.11497 | 104.2439 | 9 | Red |
| VTN621 | *Musa* | *splendida* | 28/04/2018 | Vietnam | Van Ban | Khanh Yen Thuong | 22.11497 | 104.2439 | 9 | Red |
| VTN622 | *Musa* | *splendida* | 28/04/2018 | Vietnam | Van Ban | Khanh Yen Thuong | 22.11497 | 104.2439 | 9 | Red |
| VTN623 | *Musa* | *splendida* | 28/04/2018 | Vietnam | Van Ban | Khanh Yen Thuong | 22.11497 | 104.2439 | 9 | Red |
| VTN624 | *Musa* | *splendida* | 28/04/2018 | Vietnam | Van Ban | Khanh Yen Thuong | 22.11497 | 104.2439 | 9 | Red |
